# Systemic cytokine and viral antigen-specific responses in hepatitis D virus RNA positive versus HDV RNA negative patients

**DOI:** 10.3389/fmed.2023.1125139

**Published:** 2023-10-09

**Authors:** Shivali S. Joshi, Matthew Sadler, Nishi H. Patel, Carla Osiowy, Kevin Fonseca, Carla S. Coffin

**Affiliations:** ^1^Department of Microbiology, Immunology and Infectious Diseases, Cumming School of Medicine, University of Calgary, Calgary, AB, Canada; ^2^Department of Medicine, Cumming School of Medicine, University of Calgary, Calgary, AB, Canada; ^3^National Microbiology Laboratory, Public Health Agency of Canada, Winnipeg, MB, Canada

**Keywords:** hepatitis D virus, cytokines, HBV/HDV co-infection, antigen-specific immune response, monocytes

## Abstract

**Background:**

Hepatitis B virus (HBV)/Hepatitis D Virus (HDV) co-infection increases the risk of severe liver disease compared to HBV mono-infection. Adaptive immune responses to HDV are weakly detectable, and the involvement of innate immunity in the progression of HDV-related liver fibrosis is suggested. We hypothesize that an overall innate immune activation in HBV/HDV co-infection plays a role in liver disease progression and also impacts virus specific T cell response.

**Methods:**

Sixteen HBV/HDV-co-infected-patients (median age 42y/7F/6 Asian/4 White/6 Black/15 HBeAg-) and 8 HBV monoinfected-patients (median age 39y/4F/4 Asian/3 Black/1 White/HBeAg-) with median follow-up of 5 years were enrolled. Liver fibrosis was assessed by liver stiffness measurement (LSM, FibroScan^®^). Proliferation of CD3 + CD4+ T cells in response to viral antigens using CFSE assays and cytokine secreting monocytes was analyzed by flow cytometry.

**Results:**

Of 16 HBV/HDV, 11 were HDV-RNA+ (HBV-DNA 0–1,040 IU/mL), 5/11 Interferon (IFN) + Nucleos/tide Analog (NA), 3/11 NA monotherapy, median ALT 77 U/L at the time of sample collection, median LSM of 9.8. In 5 HDV RNA−, median HBV DNA 65 IU/mL, 4/5 prior IFN and/or NA, ALT 31 U/L, and median LSM 8.5 kPa. In 8 HBV controls, median HBV-DNA, ALT, LSM was 69 IU/mL, 33 U/L,5 kPa, respectively. PBMC stimulation with HBV core antigen (HBcAg) and HDV antigen (HDAg) showed weaker CD3 + CD4 + T-cell proliferation in HDV-RNA+ vs. HDV RNA− and HBV-mono-infected patients (*p* < 0.05). In HDV-RNA+ patients, a correlation between ALT and TNF-α (*r* = 0.76, *p* = 0.008), higher IL-10 levels and increased proportion of CD14 + TNF-α+ cells were found.

**Conclusion:**

In summary, during HBV/HDV coinfection, HDV RNA+ patients had weaker HBV and HDV specific responses, associated with increased TNF-α + monocytes irrespective of IFN treatment.

## Introduction

The Hepatitis D virus (HDV) causes the most severe form of viral hepatitis in humans. It is estimated that among 296 million hepatitis B virus (HBV) surface antigen chronic hepatitis B (CHB), up to 15–20 million people are co-infected with HDV worldwide (1). HBV/HDV co-infection increases the risk of cirrhosis compared to HBV mono-infection with 70% of the co-infected cases progressing to end stage liver disease within 5–10 years (2). The underlying mechanisms of HDV induced liver disease pathogenesis are unclear. Similar to HBV, HDV is a non-cytopathic virus and the associated liver damage is thought to be an immune mediated injury (3). In chronic hepatitis D, HDV-specific adaptive response, particularly CD8+ T cell response is barely detectable and it had been recently discovered that HDV mutates to escape from the virus-specific CD8 T cell response (4, 5). One prior study found that HDV specific IFN-γ, IL-2 CD4+ T cell response is present in interferon alpha (IFN-α) induced and spontaneous viral clearance cases (5). Studies in mouse models of HDV infection and in 31 patients, have shown that innate immune responses mediated by monocytes and natural killer (NK) cells are implicated in accelerating liver damage in HBV/HDV co-infection (6–8). Further, in an immunodeficient humanized mouse model of HBV/HDV infection it was noted that co-infected mice showed increased inflammatory response which was correlated with increased liver damage compared to uninfected and HBV mono-infected mice (6). Recent studies using HBV/HDV cell culture models show that HDV but not HBV induces an innate immune response, yet the IFN induced response did not have a significant effect on HDV replication (9). Overall, these studies point out a central role of innate immune responses in HBV/HDV co-infection. We hypothesized that an overall innate immune activation in HBV/HDV co-infection drives liver damage and provide data on HDV-specific and additionally HBV specific T cell responses in co-infection.

## Patients and methods

In total 16 HBV/HDV co-infected carriers were prospectively recruited (*n* = 11/16 HDV-RNA positive) from the University of Calgary (U of C) Liver Unit. In addition, 8 HBV mono-infected cases (with similar HBV DNA levels, <2–3 log IU/mL) were enrolled. Clinical characteristics of the patients in this study are outlined in Table 1. All subjects provided informed written consent to participate according to the guidelines of the 1975 Declaration of Helsinki. This study was approved by the U of C conjoint health research ethics board, CHREB (Ethics ID 16636). Clinical data and laboratory assays such as serum HBV DNA (according to clinical PCR, Abbott Architect lower limit of detection 10 IU/mL or ~ 50 virus copies per mL) was collected. Additional HBV serology (i.e., HBsAg, HBeAg, anti-HBeAg) was determined clinically with commercial chemiluminescent microparticle immunoassays (Abbott Architect; quantitative anti-HBc II and anti-HBs). HBV genotyping was done by in-house nested PCR as previously published (10). HDV-RNA testing was performed at National Microbiology Laboratory (NML), Winnipeg. Quantitative HDV RNA was measured by real-time RT-PCR method (linear range of 3.1 log10 to 10.4 log10 copies/mL (11).

**Table 1:** Clinical characteristics of HBV/HDV and HBV patients enrolled in the study.

	HBV (*n* = 8)	HBV/HDV Gr.1 (HDV RNA+) (*n* = 11)	HBV/HDV Gr. II (HDV RNA−) (*n* = 5)
Median age in years (median IQR)	39 (18.5)	39 (14)	45 (29)
Sex	4 M/4F	5F/6M	1F/4M
Ethnicity	4 Asian 3 Black 1 White	3 Asian 4 Black 4 White	4 Asian 1 White
Median HBV-DNA (IU/mL)* [Range, median IQR]	69.5 [13–226, 164.2]	50 [0–104, 43]	65 [13–103, 58]
Median HDV-RNA (copies/mL) [Range, median IQR]	N/A	1.4×10^6^ [3.3×104-3.1×10^8^_,_ 1.3× 10^7^]	N/A
Median ALT (U/L) [Range, median IQR]	33 [8–61, 49.75]	77 [23–469, 60]	31 [12–88, 40]
Median LSM (kPa) [Range, median IQR]	5 [3.3–6.1, 3.1]	9.8 [5.3–42, 7.4]	8.5 [5.5–21.3, 9.6]
Treatment Status	Untreated	5 IFN + NA 3 NA monotherapy 3 untreated	3 IFN + NA 1 NA monotherapy 1 untreated

Gr. I: HDV-RNA+ patients, Gr. II: HDV-RNA−patients.

* 1 IU/mL = ~5.2 virus genome copies/mL.

### Isolation of peripheral blood mononuclear cells

PBMC were separated by density gradient centrifugation from ~40 mL of heparin anti-coagulated whole blood, and ~ 10^7^ cells/vial were cryopreserved. Serum isolated from 10 mL of whole blood was stored at-80oC.

### Analysis of serum cytokines

Cytokines were assessed using Human Focused 13-Plex Discovery Bead Based Immunoassay (Luminex technology) by Eve technologies, Calgary, Canada. Samples were run in duplicates. Serum and culture supernatants were used as sample types for the assay.

### Antigen specific proliferation assays

Bulk PBMC were used to perform the described assays. Fresh or cryopreserved PBMC (in 5 patients) were labeled with 1 μM carboxyfluorescein diacetate succinimidyl ester (CFSE, BD Horizon, San Diego, CA) in DPBS for 10 min at 37°C. Cells were then centrifuged at 300 g for 10 min, washed with Rosewell Park Memorial Institute (RPMI), reaction was stopped with complete RPMI (with 10% fetal bovine serum, FBS) and then suspended in complete RPMI. Labeled PBMC were stimulated with 5 μg HBsAg (adw), 5 μg HBcAg, and HDAg (American Research Products, Waltham, MA) in (RPMI) 1,640 supplemented with 10% FCS, 2 mmol/L glutamine and Pen-Strep antibiotic solution (Sigma, Oakville, ON). Phytohemagglutinin-M (PHA-M, Carlsbad, CA) 5 μg/mL or anti-CD3 (1 μg/mL)/anti-CD28 (5 μg/mL) (BD Biosciences, San Jose, CA) stimulated cells served as positive control. Unstimulated DMSO treated cells were negative controls since CFSE was dissolved in DMSO. Cells were cultured in triplicates and plates were incubated at 37°C with 5% CO_2_ for ~7 days. Cell proliferation was assessed on day 7 or 8. Stimulation index (SI) was calculated as % CFSE low cells in stimulated cells / % CFSE low cells in the unstimulated (DMSO) control as per our previously established protocols (12). SI values >2 were considered as positive for antigen specific proliferation. Supernatants were collected at 72 h, stored in-80°C and analyzed in a Luminex assay to study cytokine levels.

### Cytokine release by monocytes

Approximately 10^6^ fresh PBMC were stimulated with 5 μg/mL lipopolysaccharide (LPS, Sigma, Oakville, ON) for 18 h. Unstimulated DMSO treated cells were used as negative controls. After 2 h, 1 μg/μL per million cells Golgi-Plug was added. After 16 h, cells were washed and stained with FVS510 to exclude dead cells and with anti CD14-BV605 to identify monocytes. Cells were washed again, fixed, and permeabilized with the Cytofix/Cytoperm Kit, stained with PE conjugated antibodies against TNF, IL-1β, IL-10 for 30 min at 4^o^ C, washed, re-suspended in PBS, and immediately analyzed by flow cytometry. A positive response was defined as >2 fold the DMSO background response. All antibodies and reagents used for flow cytometry were purchased from BD Biosciences, Mississauga, ON unless mentioned otherwise.

### Statistical analyses

Data were analyzed using Graphpad Prism 7 (La Jolla, CA). Demographic, clinical and laboratory parameters were compared using measures of central tendency, Mann–Whitney U test and Kruskal-Wallis test with post-hoc Dunn’s test for multiple comparisons was used to compare flow data. Non-parametric Spearman’s rank correlation test was used for correlation analysis; *p* values <0.05 were considered significant.

## Results

### Summary of clinical data

In total, 16 HBV/HDV co-infected cases were enrolled in the study. Of these *n* = 11 were HDV-RNA positive at the time of sample collection with 5 on IFN/NA combination, 3 on NA monotherapy and 3 untreated (HBV/HDV Group I, HDV-RNA+ median HDV-RNA 1.4×10^6^ copies/mL). In comparison, 5/16 had undetectable HDV-RNA (HBV/HDV Group II, HDV-RNA−) with 3 previously treated with IFN + NA therapy, 1 on NA monotherapy and 1 untreated. It is known that co-existence of HBV and HDV, usually results in HBV suppression and HDV dominance (13). In our cohort of HBV/HDV co-infected individuals, median HBV levels were ~ 60 copies/mL, thus as a control, 8 HBV mono-infected patients (HBV) with similar HBV-DNA levels (69 copies/mL) were enrolled (see Table 1, Supplementary Table 1).

### Estimation of serum cytokines and correlation to alanine transaminase levels

**Figure 1:**
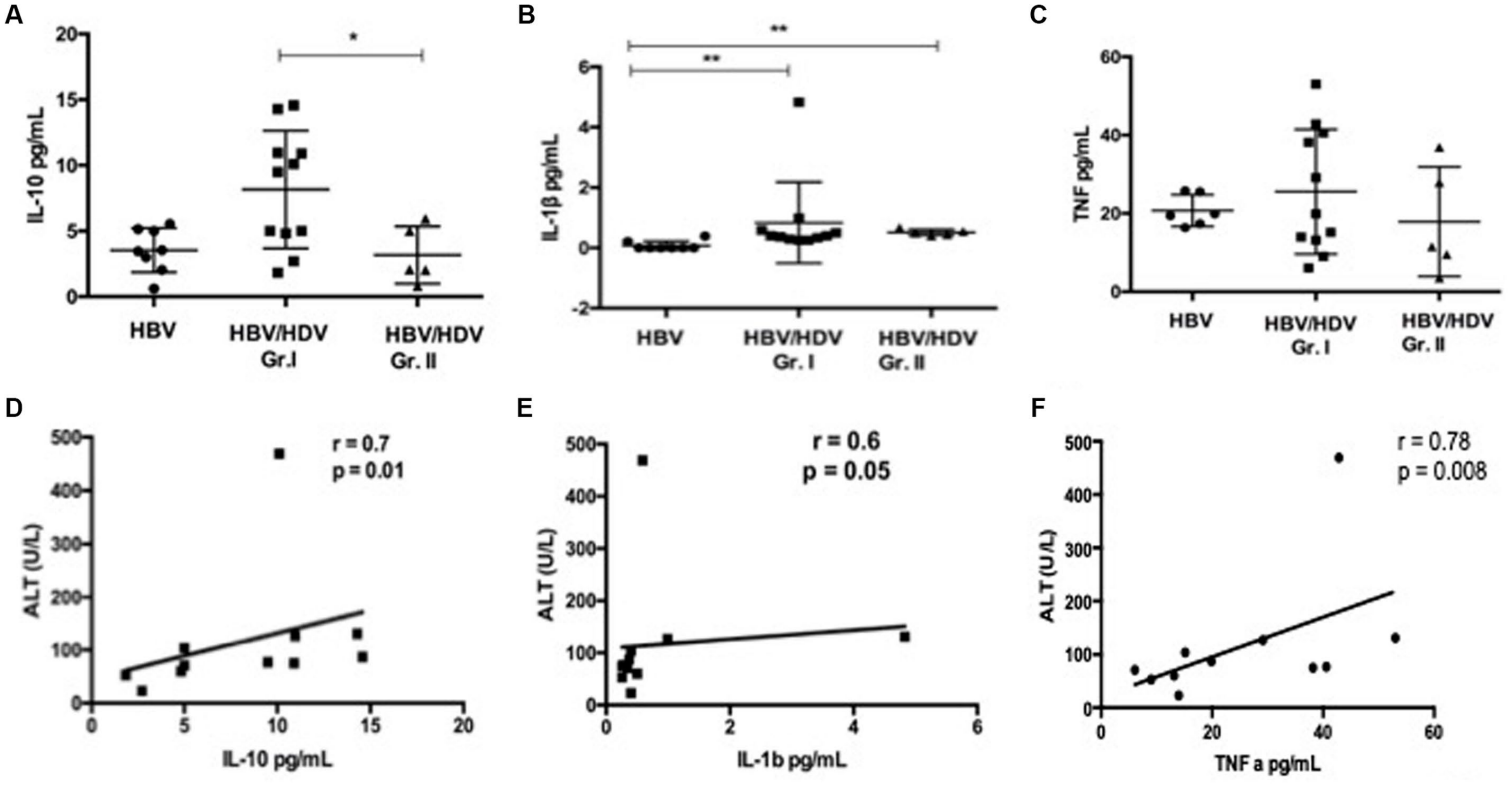
Comparison of serum cytokines in HDV patients **(A)** IL-10, **(B)** IL-1β, and **(C)** TNF-α in pg./mL in HBV/HDV Gr. I (HDV RNA+) vs. HBV/HDV Gr. II (HDV RNA−) patients. Correlation analysis between ALT and serum cytokines, **(D)** IL-10, **(E)** *IL*-1β, and **(F)**. TNF-α in HDV RNA+ patients. * *p* < 0.05, Kruskal–Wallis test.

We tested 13 pro-inflammatory, Th1 and Th2 cytokines in co-infected and mono-infected patients. Of note, IL-10 was significantly increased in the HDV-RNA+ group compared to the other groups (Figure 1A). We observed an increase in IL-1β in HBV/HDV co-infection vs. HBV mono-infection (*p* < 0.05) (Figure 1B). A similar trend was also noted for TNF-α (Figure 1C). Interestingly, all of the 13 cytokines studied were elevated although non-significant increase in the HDV-RNA+ patients compared to HDV-RNA− and HBV mono-infected groups (Table 2). Several studies have studied cytokines as biomarkers of liver disease. We therefore performed correlation analysis between ALT and each of the cytokines. Of all the soluble markers investigated, TNF-α, IL-1β, and IL-10 were significantly and positively correlated with ALT levels (Figures 1D–F).

**Table 2:** Serum cytokines and chemokines in HBV/HDV co-infection and HBV mono-infection.

	HBV	HBV/HDV Gr. I (HDV RNA+)	HBV/HDV Gr. II (HDV RNA−)
IFN-ץ	1.3 ± 0.2	5 ± 3.8	1.4 ± 0.2
IL-2	0.58 ± 0.12	1.9 ± 0.9	0.6 ± 0.04
IL-4	1.9 ± 0.49	3.8 ± 1.9	1.07 ± 0.3
IL-5	0.15 ± 0.06	0.8 ± 0.27	0.4 ± 0.03
IL-6	0.6 ± 0.16	0.8 ± 0.4	0.47 ± 0.19
IL-8	4 ± 0.9	32 ± 13.63	9.5 ± 1.3
IL-12	0.4 ± 0.18	3.4 ± 2.4	0.4 ± 0.2
IL-13	0.63 ± 0.16	0.89 ± 0.44	0.47 ± 0.19
GM-CSF	3 ± 0.33	13.65 ± 8.7	3.5 ± 0.3
MCP	2.1 ± 0.89	16.6 ± 10	0.8 ± 0.2

### Cytokine production by monocytes

**Figure 2:**
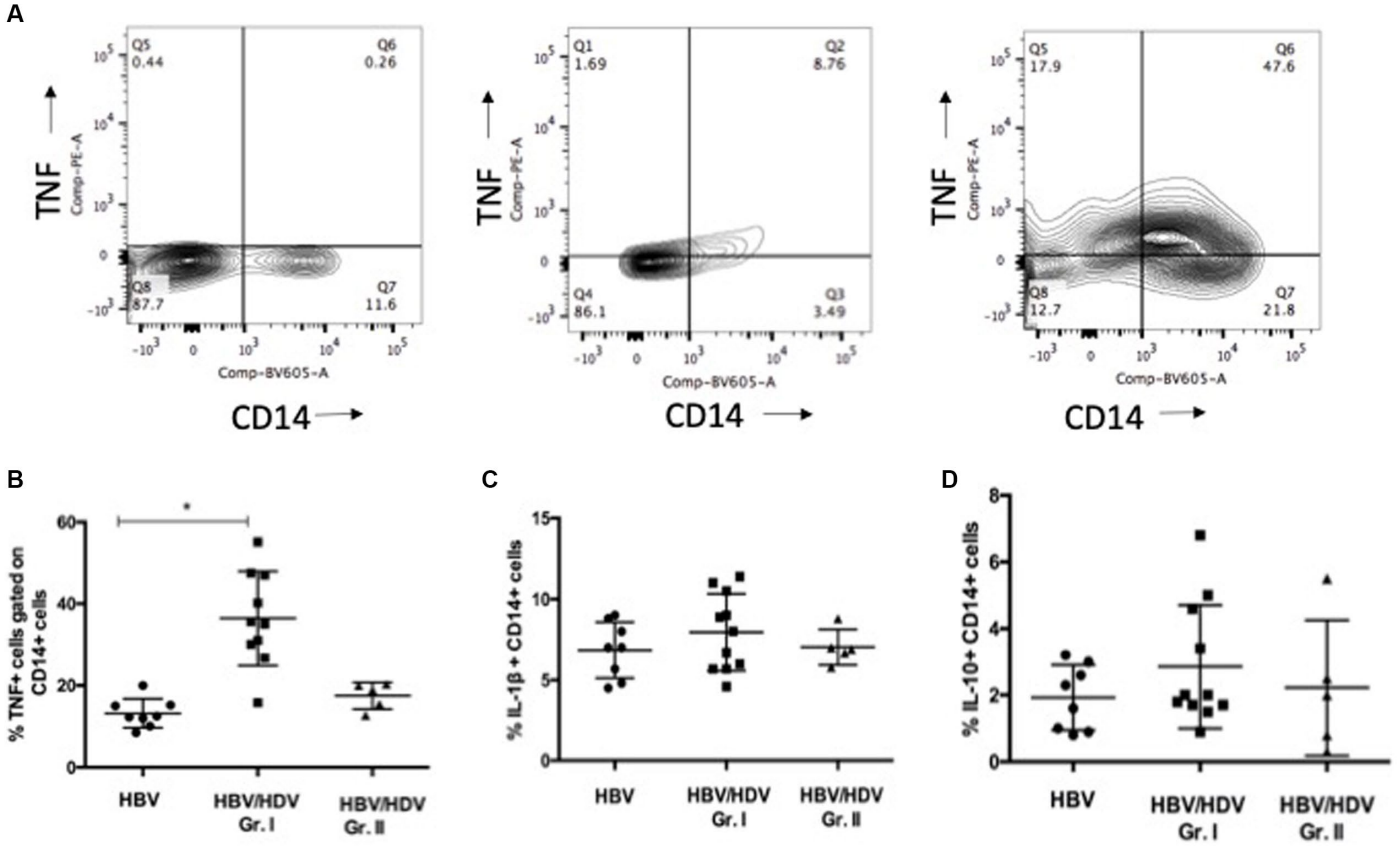
Comparison of CD14+ TNF+, CD14 + IL-1β, CD14 + IL-10+ monocytes in HBV/HDV Gr. I vs. other groups. **(A)**. Representative plots of CD14 + TNF+ cells in negative control (DMSO control), HBV/HDV Gr. II (HDV RNA+) and HBV/HDV Gr. I (HDV RNA −) patients. **(B)**. Frequency of CD14 + TNF+ cells. **(C)**. Frequency of CD14 + IL-1β cells. **(D)**. Frequency of CD14 + IL-10+ cells in the three categories. Individual values have been plotted. * *p* < 0.05, Kruskal–Wallis test.

A previous study highlighted the role of Inducible Protein (IP) - 10 producing monocytes from peripheral blood and CD206+ macrophages in the liver in HBV/HDV co-infection and liver fibrosis and/or inflammation (5, 7). Further, monocyte activation and cytokine release is implicated in viral hepatitis and viral co-infections (14, 15). Thus, we explored activation induced (LPS) cytokine secretion by CD14+ monocytes. Although, no difference was noted for IL-10 and IL-1β, a significant increase in the proportion of CD14 + TNF+ PBMCs was noted in the HDV-RNA+ group vs. – mono-infected group and a similar trend was noted for HDV-RNA+ vs. HDV-RNA−group (Figures 2A–D).

### HBV and HDV specific CD4 T-cell response in HBV/HDV co-infection using bulk PBMC

**Figure 3:**
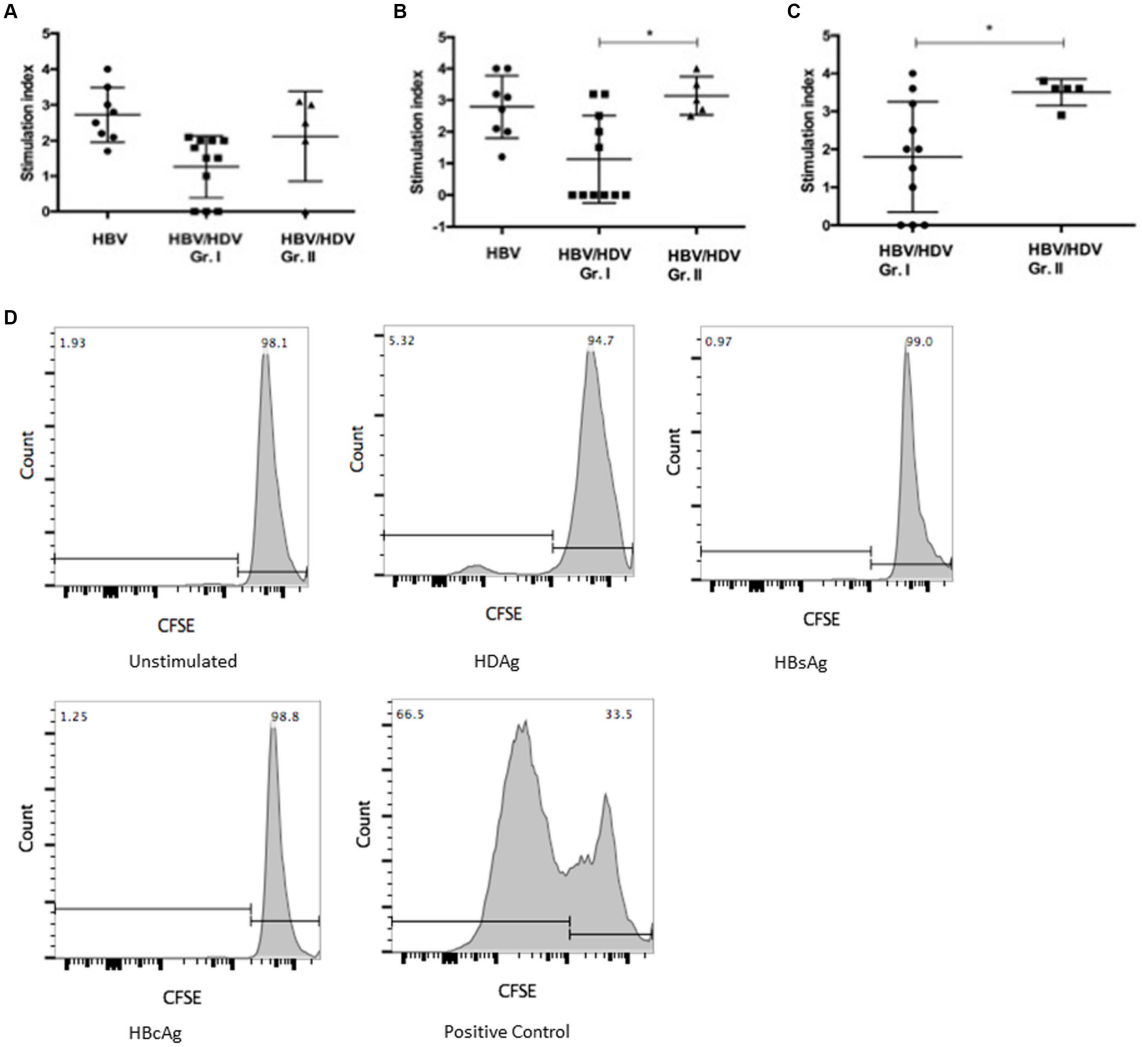
Comparison of T-cell proliferation in PBMC from HBV/HDV co-infected patients and HBV mono-infected patients in response to viral antigen stimulation **(A)** HBsAg, **(B)** HBcAg, and **(C)** HDAg specific CD3 + CD4+ T-cell response in HBV/HDV Gr. I (HDV RNA+) vs. HBV/HDV Gr. II (HDV RNA−) patients. * *p* < 0.05, Kruskal–Wallis test and Mann–Whitney test. Values represent mean ± standard deviation. **(D)** Representative CFSE proliferation prolife in a HDV-RNA+ patient (HBV/HDV Gr. I) in response to unstimulated, HDAg, HBsAg, HBcAg, and mitogen stimulated positive control.

We hypothesized that presence of activated monocytes and systemic inflammation contributes to enhanced T-cell responses (16). We examined HBsAg, HBcAg, and HDAg specific responses *ex vivo* using PBMC from co-infected and mono-infected individuals. Surprisingly, we found reduced HBcAg and HDAg specific proliferation of CD4+ T cells in HDV-RNA+ patients compared to HDV-RNA−group (*p* < 0.05) (Figures 3A–D).

### Th1 and Th2 cytokines in PBMC culture supernatants

Levels of IL-2, IL-4, IL-5, TNF-α, and IFN-γ in the PBMC culture supernatants from HDV-RNA+ cases were lower than in HDV-RNA−and HBV mono-infected cases, especially in response to HBcAg and HDAg. In 8/11 HDV-RNA+ patients, Th1 cytokines – IL-2, IFN-γ were undetectable in response to HBcAg (Table 3).

**Table 3:** Th1 and Th2 cytokines in HBV/HDV co-infection and mono-infection in response to HBV and HDV proteins *in-vitro*.

HBsAg	IL-4 (pg/mL)	IL-5 (pg/mL)	IL-2 (pg/mL)	IFN- ץ (pg/mL)	TNF-α (pg/mL)
HBV/HDV Gr. I	4.5 (3.4)	6 (0.8)	2.8 (1)	8.1 (2)	10.2 (0.9)
HBV/HDV Gr. II	5.4 (0.6)	7.2 (1)	2.5 (1.4)	5.6 (0.2)	11.5 (0.5)
HBV	4.6 (1)	8.4 (1.1)	3 (0.6)	9.6 (1.1)	10.9 (2.5)

HBcAg	IL-4	IL-5	IL-2	IFN- ץ	TNF-α
HBV/HDV Gr. I	2.5 (0.2)	5.5 (0.4)	2.4 (2.3)	3.5 (4.4)	15 (3.9)
HBV/HDV Gr. II	4.7 (0.6)	8 (1.9)*	5.1 (2)*	7.8 (2.2)*	12.5 (2.5)
HBV	3.5 (0.5)	4.2 (1)	3.5 (0.7)	2.1 (0.9)	11.4 (3.5)

HDAg	IL-4	IL-5	IL-2	IFN- ץ	TNF-α
HBV/HDV Gr. I	3.5 (1.2)	2 (0.8)	2.8 (1)	1.9 (0.6)	9 (1.2)
HBV/HDV Gr. II	6.7 (1)*	5.7 (1.1)*	6.2 (0.4)*	5 (1)**	13 (2.2)

*p < 0.05, ** <0.01 by Kruskal–Wallis test.

Values in parentheses represent standard deviation. Values represent pg/mL of cytokines stimulated – unstimulated culture supernatants.

### Summary of antigen specific response in the 16 HBV/HDV co-infected patients

Based on cytokine release in antigen stimulated culture supernatants and proliferation experiments all the HDV-RNA negative cases (*n* = 5) showed HDV and HBV (especially HBcAg) specific T cell responses analyzed in bulk PBMC. 6/11 HDV-RNA positive cases did not show responses to either HBV or HDV antigens. These results show differences in viral antigen specific immune response in HDV-RNA positive vs. negative patients. Table 4 reports T cell responses for *n* = 16 HBV/HDV co-infected patients.

**Table 4:** Virus specific T-cell response in patients with HBV/HDV co-infection.

Sample ID #	Date of sample collection	HDV RNA status at the time of sample collection	Response to HDAg	Response to HBsAg	Response to HBcAg
103	January 2017	Negative	Yes	Yes	Yes
104	January 2017	Negative	Yes	Yes	Yes
177	May 2017	Positive	No	No	No
218	June 2018	Negative	Yes	No	Yes
323	July 2016	Positive	No	Yes	No
325	May 2017	Positive	No	No	No
332	May 2017	Positive	Yes	Yes	Yes
336	January 2017	Positive	No	No	No
342	August 2018	Negative	Yes	Yes	Yes
347	May 2017	Positive	Yes	Yes	Yes
353	July 2017	Positive	Yes	No	Yes
358	August 2017	Positive	Yes	No	Yes
360	October 2017	Positive	No	No	No
364	October 2017	Positive	No	No	No
370	December 2017	Positive	No	No	No
395	August 2018	Negative	Yes	Yes	Yes

## Discussion

Chronic hepatitis D is the most severe form of viral hepatitis with very limited treatment options and poorly understood immunological aspects of disease progression (3). In this study, we show that an increased inflammatory response was observed in “active” HDV-RNA+ patients compared to HDV-RNA−cases in association with weak HBV and HDV specific T-cell response, irrespective of IFN treatment history.

The current data suggests that in cases with HDV viremia, an overall immune activation exists. Further, TNF-α, IL-1β, and IL-10 levels correlated with ALT suggesting a role in liver damage in the setting of co-infection. Townsend et al. also reported increased systemic cytokines in the HDV patients with a high viral load (6.1–6.4 HDV-RNA, IU/mL) vs. mild HDV cases (1.5–6 IU/mL) (17). A previous study in an immuno-deficient mouse model of HBV/HDV co-infection showed high levels of basal pro-inflammatory and pro-fibrogenic cytokine levels which may directly contribute to liver disease progression (6). Interestingly, in an adenovirus HBV/HDV mouse model, the use of a TNF-α agonist resulted in significant reduction in HDV related liver damage (18). We noted an increased proportion of TNF producing monocytes in response to LPS treatment in HDV-RNA+ patients. Another study reported IP-10 release from monocytes when stimulated with HDV peptides (5). In human liver tissue (*n* = 15), intrahepatic CD14+ cells were found to be associated with pathological inflammation (HBV, HBV/HDV, HCV). Intrahepatic leucocytes from these livers produced high levels of TNF-α, IL-1β and IL-6 compared to healthy livers (7). Ito et al. elegantly proved in HBsAg transgenic/TNF double knockout mice that TNF-α produced by intrahepatic non antigen specific inflammatory cells is critical in the development of lethal liver disease (19). Taken together, these findings highlight the role of pro-inflammatory monocytes in liver injury and also suggest that TNF+ monocytes may be a potential biomarker in predicting risk of liver disease in HBV/HDV co-infection. In chronic hepatitis C, a decrease in LPS stimulated TNF production by monocytes was linked to poor disease outcome (20). In contrast, an increase in PBMC CD11b + macrophage frequency and phagocytic activity has been reported in fulminant hepatitis E (21). Decreased T-cell response in association with LPS activated monocytes has been previously reported. The state of inflammation in HDV-RNA+ patients is similar to the systemic inflammatory milieu in obesity (i.e., enhanced TNF levels and also conversion of memory T cells to naïve T cell subsets) (22, 23). These reports and our data point out toward differential hepatitis D virus specific monocyte functions.

HBV/HDV is a dynamic disease with fluctuating patterns of HBV and HDV dominance over time. Thus, it is crucial to study HBV specific response as well as HDV specific immune response. We found weak HBsAg and HBcAg specific CD4+ T-cell proliferation in HDV-RNA+ cases vs. HDV-RNA−and HBV mono-infected patients. Similarly, reduced HDV specific response was noted in HDV-RNA positive vs. negative cases. Nisini et al. found that PBMC from 8 of 30 patients (27%) significantly proliferated in response to HDAg. Another study showed that 12/46 (28%) Studies have shown that ~28% (8/30 and 12/46)of HBV/HDV patients show proliferation in response to HDAg (24, 25). Prior immunologic studies in CHB showed an increase of HBcAg/HBeAg-specific T cell proliferation before and during ALT flares along with an increased production of Th1 cytokines IFN-γ and IL-2 was noted (26, 27). In our study, we found that Th1 response was barely detectable in HDV-RNA+ cases despite ALT flares. These data suggest a differential regulation of T-cell response in HBV/HDV co-infection than mono-infection. Severe liver damage in HBV/HDV co-infection in chimpanzees correlated with a lack of Th1 response (28). Similar to Grabowski et al., we noted that HDV-specific IFN-γ and IL-2 responses were detectable in all HDV-RNA−patients and 4 HDV-RNA+ patients after *in vitro* stimulation of PBMC with HDAg (5). It would be interesting to note the dynamics of T-cell response in serial samples collected from HDV-RNA positive patients to gain further insights on viral clearance and viral relapse in HDV-RNA negative cases. Sorting and co-culture of monocytes and T cells to clearly delineate the role of monocytes in modulating T-cell mediated response in HBV/HDV co-infection.

The current single center study is limited by small sample size of 16 HBV/HDV co-infected patients (*n* = 11 HDV RNA+ and 5 HDV RNA−), and inability to assess intrahepatic immune response with liver biopsy. Hepatitis Delta is an orphan disease and there is limited clinical study on immunological aspects of HBV and HDV mediated liver fibrosis progression. A German study of 41 HDV/HBV patients showing chronic HDV infection engages the mucosal-associated invariant (MAIT) T cell compartment causing activation, functional impairment, and subsequent progressive loss as the potential cause of HDV-associated liver disease progresses, although the study could not link this data to long-term clinical outcomes (29). The authors hypothesized that cytokine driven (IL-12 and IL-18) activation could lead to cell death and peripheral loss of MAIT cells, like other inflammatory conditions including autoimmune hepatitis. Landahl et al., analyzed HDV specific T cell responses at single peptide level in 32 HDV infected patients in Italy (vs. 6 monoinfected) following *in-vitro* stimulation with 21 overlapping peptides, and found >1 T-cell response in 50% tested but no difference in HDV RNA positive vs. negative patients (30). A study by Kefalakes et al., found that in 28 HBV/HDV co-infected patients activated but not terminally differentiated HDV-specific CD8 T-cell response correlated with liver disease progression. Furthermore, in half of the patients, HDV clearance by CD8 T cells did not occur and reduced T cell activation in association with escape variants of HDV was noted (31). Loss of cytokine production and proliferation has been attributed to T cell exhaustion in HCV and HBV infections but is widely understudied in HBV/HDV co-infections and may be an area for future investigation in our HBV/HDV patient cohort (32).

## Conclusion

There is limited data on immunological aspects of HBV and HDV mediated liver fibrosis progression. The current study provides further detail characterization of the functionality of HBV and HDV specific T cell responses and cytokine responses compared to HBV monoinfection, linked to long-term clinical and virological outcomes. Hepatitis Delta co-infected patients with viremia show higher activation induced TNF alpha release by monocytes in association with weak HBV and HDV specific T-cell responses compared to HDV-RNA negative patients irrespective of anti-viral treatment status. The study contributes to limited data on immunopathogenesis of hepatitis Delta virus Infection.

## Data availability statement

The original contributions presented in the study are included in the article/Supplementary material, further inquiries can be directed to the corresponding author.

## Ethics statement

The studies involving humans were approved by the University of Calgary Conjoint Health Research Ethics Board, CHREB (Ethics ID 16636). The studies were conducted in accordance with the local legislation and institutional requirements. The participants provided their written informed consent to participate in this study.

## Author contributions

SSJ: study design, execution, data analysis, and preparation of manuscript. CSC: study design, manuscript draft, and patient recruitment. MS: patient recruitment. NHP: data analysis, manuscript review, and feedback. CO and KF: HDV genotyping data. All authors contributed to the article and approved the submitted version.
